# Marine yeast isolation and industrial application

**DOI:** 10.1111/1567-1364.12158

**Published:** 2014-05-21

**Authors:** Abdelrahman Saleh Zaky, Gregory A Tucker, Zakaria Yehia Daw, Chenyu Du

**Affiliations:** 1 School of Biosciences, University of NottinghamNottingham, UK; 2 Department of Microbiology, Faculty of Agriculture, Cairo UniversityGiza, Egypt

**Keywords:** marine yeast, isolation, biofuel, enzyme, pharmaceuticals, seawater

## Abstract

Over the last century, terrestrial yeasts have been widely used in various industries, such as baking, brewing, wine, bioethanol and pharmaceutical protein production. However, only little attention has been given to marine yeasts. Recent research showed that marine yeasts have several unique and promising features over the terrestrial yeasts, for example higher osmosis tolerance, higher special chemical productivity and production of industrial enzymes. These indicate that marine yeasts have great potential to be applied in various industries. This review gathers the most recent techniques used for marine yeast isolation as well as the latest applications of marine yeast in bioethanol, pharmaceutical and enzyme production fields.

## Introduction

Marine microorganisms have potential applications in metal detoxification, nutrient cycling, greenhouse gas reduction and form the basis of food webs. Marine microorganisms, including marine yeasts, live in extreme environments, and this provides a unique potential for the synthesis of functional biomolecules (Connell *et al*., 2008). Marine yeasts, defined as the yeasts that are isolated from marine environments, are able to grow better on a medium prepared using seawater rather than freshwater (Chi *et al*., 2010). The first marine yeasts were isolated by Bernhard Fischer in 1894 from the Atlantic Ocean, and those were identified as *Torula* sp. and *Mycoderma* sp. (Kutty & Philip, 2008). Following this discovery, various other marine yeasts have been isolated from around the world from different sources, including seawater, seaweeds, marine fish and mammals as well as seabirds. Among these isolates, some marine yeasts originated from terrestrial habitats (grouped as facultative marine yeast), which were brought to and survived in marine environments. The other marine yeasts were grouped as obligate or indigenous marine yeasts, which confine to marine habitats (Kohlmeyer & Kohlmeyer, 1979; Kutty & Philip, 2008). However, no sufficient evidence has been found to explain the indispensability of seawater for obligate marine yeasts. It has been reported that marine yeasts are able to produce many bioactive substances, such as amino acids, glucans, glutathione, toxins, enzymes, phytase and vitamins with potential application in the food, pharmaceutical, cosmetic and chemical industries as well as for marine culture and environmental protection (Chi *et al*., 2009; Sarkar *et al*., 2010).

## Marine yeast isolation methods

Over the years, microbiologists have developed several methods for marine yeast isolation. These methods differ in their sampling, sample preparation, medium composition and strain maintenance. This variation is required to cope with the diverse marine habitats, the target properties required in the isolates (e.g. the ability of utilizing xylose) and the likely cell density of the sample.

Surface seawater samples can be collected using simple plastic or glass bottles (1–5 L). Bottles should have screw caps for easy handling as well as for preventing contamination and leaks. For aseptic reason, bottles should be opened under water and washed thoroughly using the seawater 3–5 times before filling with sample. Sterilized plastic bags, jars and vials can also be employed in collecting surface samples. Surface seawater samples are suitable for isolating aerobic and facultative anaerobic yeasts. A ‘near shore’ location is more suitable for sampling yeasts that are capable of carbohydrate fermentation (Fell, 2001). Samples of 250 mL are generally enough when they are taken near shore, whereas samples from the open ocean should be at least 1 L as a lower microorganism density is expected. Fifty millilitres of sediment samples is generally considered adequate. Experiment design and replication should be taken into account for the required sample volume (Fell, 2001).

**Figure 1:**
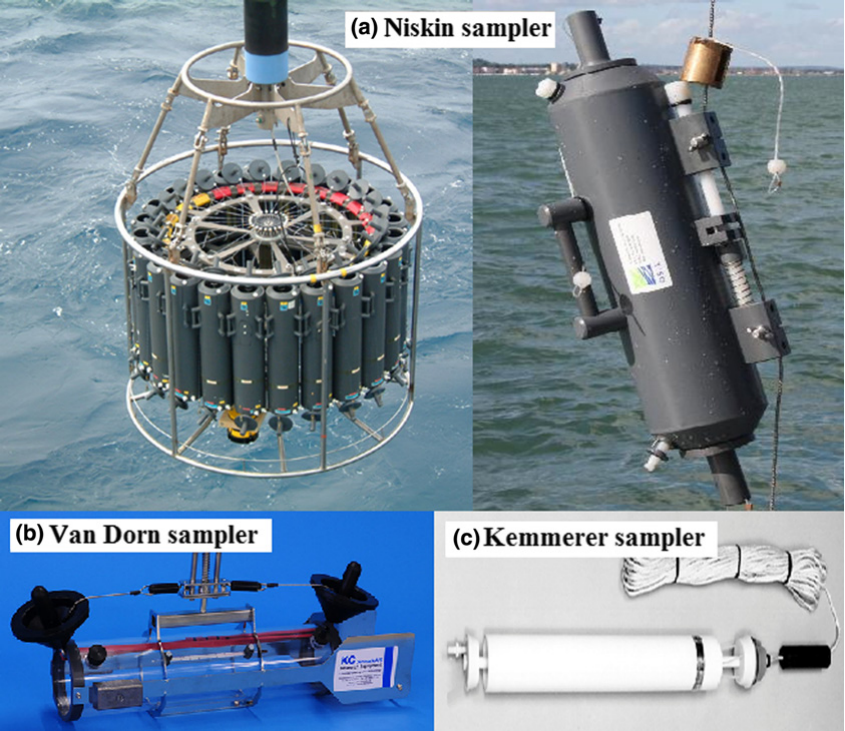
The commonly used samplers for deep seawater sampling. (a) Niskin sampler (http://www.godac.jamstec.go.jp/darwin/instrument/mirai/e), (b) Van Dorn sampler (http://www.kc-denmark.dk/products/water-sampler/van-dorn-water-sampler.aspx) and (c) Kemmerer sampler (http://www.rickly.com/as/kemmerer.htm).

More advanced devices have been designed and used to collect deep sea samples (water and sediments). The first water sampler that was able to maintain *in situ* hydrostatic pressure was reported by Jannasch *et al*. (1973). Generally, Niskin, Van Dorn and Kemmerer samplers are the most common apparatuses that have been used for deep sea sampling, as shown in Fig.1. Niskin samplers can be used singly or in series or in a rosette of up to 12 samplers per rack. Van Dorn is a horizontal sampler, while Kemmerer is a vertical sampler so that it could fit narrow areas. These devices can collect samples from as deep as 6000 m. However, those devices do not maintain *in situ* hydrostatic pressure. These samplers usually consist of cylindrical tube(s) with a stopper at each end (between 1 and 121 tubes per frame). These stoppers could be controlled remotely from the surface (Dorschel, 2007; Singh, 2011). Research submarines can also be used to collect deep sea samples. This device is larger in size, very complicated and massively expensive. On the other hand, research submarines allow the collection of large amounts of samples, good observation of the sample environment and instant work on the samples as it can also carry all the laboratory equipment needed (Singh, 2011).

Sample preparation for marine yeast isolation is depended on two main factors (1) the desired characteristics of the isolates and (2) the expected number of yeast cells per millilitre. Samples collected from the open sea usually contained around 10 or fewer cells per millilitre (Fell, 2001; Kutty & Philip, 2008). Therefore, filtration of 5 L seawater is required followed by the resuspension of the cells remaining on the filter in 15 mL of the same seawater filtrate. In contrast, samples collected from the high organic matter containing surface near shore can contain thousands of yeast cells per millilitre (Kutty & Philip, 2008). So, filtration of 100 mL is usually enough. Alternatively, samples could be subject to an enrichment step for a couple of days before isolation to select desirable strains with specific characteristics. For extraction from solid samples, such as seaweed, sea sand, dead marine plant and animal material, a known weight of solid particles can be transferred into a broth medium for enrichment, or be placed directly on an agar plate. Serial dilution is required prior to isolation if more than 300 isolates are expected per millilitre (Fell, 2001).

Several different medium recipes have been used for the isolation of marine yeasts. Although both natural and artificial seawater have been used for preparing medium, natural seawater is preferable as it is closer to the natural environment that the yeasts inhabit. A mixture of broad-spectrum antibiotics have been used in isolation media, which have been shown to be more effective than single antibiotic in inhibiting the growth of bacteria and were less harmful to yeast cells (Beuchat, 1979; Thomson, 1984). Different inhibitors, including rose Bengal (Jarvis, 1973; King *et al*., 1979), dichloran (Jarvis, 1973) and propionate (Bowen & Beech, 1967) have been added to the media to inhibit the growth of moulds (Kutty, 2009). Usually, the same medium as used for isolation is also used for maintenance but without added antibiotics. Plates should be incubated at a temperature similar to the environment where the samples were collected. The optimum temperature for marine yeasts varies (Watson, 1987). For taxonomic tests, yeasts are usually incubated at 25 °C (Buhagiar & Barnett, 1971). The following list gives some media and incubation conditions suggested by researchers for the isolation of marine yeasts:

1. Wickerham's yeast malt medium (Wickerham, 1951): This medium is widely used for marine yeast isolation. It contains (w/v) 1% glucose, 0.3% yeast extract, 0.3% malt extract, 0.5% peptone and 2% agar. All the chemicals are prepared in seawater at a salinity equivalent to the sample site. About 200 mg L^−1^ of chloramphenicol was added into the medium prior to autoclaving and the final pH was adjusted to 7.0. Alternatively, an antibiotic mixture of penicillin G and streptomycin sulphate (each at 150–500 mg L^−1^) can be added to the autoclaved, cooled (below 45 °C) medium.
2. Chi *et al*. (2007) modified a liquid YPD Medium (2.0% glucose, 2.0% polypeptone and 1.0% yeast extract, w/v) by preparing the medium with natural seawater instead of fresh water. 0.05% (w/v) chloramphenicol was also added. This medium should be prepared immediately after sampling and cultivated at the natural temperature for 5 days.
3. Wang *et al*. (2007) prepared a seawater nutrient agar medium consisting of (w/v) 2.0% glucose, 2.0% peptone, 1.0% yeast extract, 2.0% agar. Components were dissolved in half-strength artificial seawater, and the pH of the medium was then adjusted to 4.5. The agar plates were incubated for 5 days at 20 °C. The composition of the artificial seawater was (per litre) as follows: NaCl, 20 g; KCl, 0.35 g; MgCl_2_·6H_2_O, 5.4 g; MgSO_4_·7H_2_O, 2.7 g; CaCl_2_·2H_2_O, 0.5 g.
4. Masuda *et al*. (2008) used an YPD agar, containing (w/v) 2.0% glucose, 2.0% peptone, 1.0% yeast extract and 2.0% agar, supplemented with 3.0% NaCl and 100 ng μL^−1^ of chloramphenicol at pH 6.0. The plates were incubated at 25 °C for 5–7 days.
5. Hernandez-Saavedra *et al*. (1995) used an isolation medium consists of (w/v) 2.0% glucose, 1.0% peptone, 5.0% yeast extract and 2.0% agar prepared in filtered seawater. The pH was adjusted to 4.5 with 0.1 N HCl.
6. Loureiro *et al*. (2005) used a modified Sabouraud's dextrose agar medium (0.5% peptic digest of animal tissue, 0.5% pancreatic digest of casein, 4.0% dextrose and 1.5% agar, w/v). They added yeast extract and chloramphenicol to the medium and incubated the plates at 28 ± 1 °C. The concentrations of yeast extract and chloramphenicol were not reported.
7. Dinesh *et al*. (2011) used Sabauroud's dextrose agar medium prepared in 50% seawater. The plates were incubated at 35 °C for 48 h.
8. Nagahama *et al*. (1999) prepared an YM agar medium from Difco, which was dissolved in artificial seawater (3% NaCl, 0.07% KCl, 1.08% MgCl_2_, 0.54% MgSO_4_, 0.1% CaCl_2_, w/v). This was then used for isolating yeast from a cold marine habitat. Medium was then supplemented with 0.01% (w/v) chloramphenicol and 0.002% (w/v) streptomycin. The plates were incubated at a low temperature (5–10 °C) for 2 weeks and then at 20 °C for 1 month.
9. Sarlin and Philip (2011) suggested a malt extract agar medium containing (w/v) 2.0% malt extract, 0.5% mycological peptone and 2.0% agar. It was suspended in around 50% diluted seawater at pH 6.
10. Kodama (1999) described a medium consisting of (w/v): sucrose 20%, polypeptone 3%, yeast extract 0.3%, chloramphenicol 100 mg L^−1^, agar 1.5%. The medium was prepared using filtered seawater, and the pH was adjusted to 5.6.
11. Khambhaty *et al*. (2013) suggested a method combining filtration followed by enrichment before isolation. The enrichment medium was GYP broth consisting of (w/v): glucose 1%, peptone 0.5%, yeast extract 0.5% and sodium chloride 2.5%. Enrichment was carried out for 24 h at 30 °C in a shaking incubator. A loopful of the suspension was spread on GYP plates consisting of (w/v): glucose 1%, peptone 0.5%, yeast extract 0.5%, sodium chloride 2.5% and agar 2.5%. The plates were incubated at 30 °C for 2–3 days.
12. We have recently developed a three-step method for marine yeast isolation. In the first step, samples were subjected to three cycles of enrichment using a nutrient rich media supplemented with a mixture of antibiotics (penicillin G 500 mg L^−1^ and streptomycin sulphate 500 mg L^−1^). The medium in the first and second cycles consist of 3.0% glucose, 3.0% xylose, 0.3% malt extract, 0.3% yeast extract, 0.5% peptone, 0.1% (NH_4_)_2_SO_4_, 0.05% KH_2_PO_4_. The medium composition for the last cycle was 6.0% of the desired carbon source (e.g. glucose or xylose or galactose), 0.3% yeast extract, 0.5% peptone, 0.1% (NH_4_)_2_SO_4_, 0.05% KH_2_PO_4_. All media were prepared using natural seawater and adjusted to pH 5. Each cycle of enrichment was carried out in a shaking incubator at 150 r.p.m., 30 °C for 48 h. In the second step, the culture from the final cycle of enrichment was incubated at 30 °C for 24–48 h on agar plates. In the third step, single colonies from step 2 were transfer to new agar plates and were checked for their morphology under the microscope.

The above-mentioned incubation conditions, including temperature, were suggested by the researchers and could be changed according to the experiment requirement. The growth on plates should be observed daily. Parts of any suspected yeast colonies should be picked up and transferred onto a microscope slide for inspection. Streak plate technique should be applied on confirmed yeast colonies using YPD seawater agar medium without antibiotics. Streak plate should be repeated to ensure the purity of the isolate. Colonies of interest can be transferred into a slant culture tube for further study.

## Use of marine yeast for bioethanol production

Bioethanol and biodiesel are two important liquid biofuels. Bioethanol production has been increased worldwide gradually in the last decade to around 85 billion litres in 2011 (Pensupa *et al*., 2013). Due to the increasing demand on energy with an ever growing in world population and the limited supply of fossil fuels, the contribution of bioethanol is expected to increase further over the next decades. Currently, bioethanol production is carried out using media containing freshwater and terrestrial yeast strains. In the fermentation step, bioethanol production consumes 2.7–5.8 gallons of freshwater per gallon of bioethanol produced when corn is used as the carbohydrate substrate (Wu & Chiu, 2011). In a cellulosic to bioethanol process, it has been estimated that between 4.5 and 5.3 gallons of freshwater would be consumed to produce one gallon of bioethanol (Wu & Chiu, 2011). Seawater, which accounts for 97% of the world's water, can be a promising alternative water resource for bioethanol production in coastal cities, especially in the Middle East and other arid zones where fresh water is increasingly precious. Additionally, seawater contains a spectrum of minerals and as such may avoid the addition of essential nutrients currently required for commercial fermentation medium (Lin *et al*., 2011). Thus, using seawater in fermentations could potentially improve the overall economics of the process by both reducing the freshwater intake and producing freshwater through distillations in the biorefinery. Therefore, the development of seawater-based bioethanol strategies can certainly make a strong impact on overcoming both the fresh water and energy crises.

Over the last few decades, halo-tolerant yeasts have been investigated as promising alternative candidates for bioethanol production. Urano *et al*. (2001) isolated several marine yeasts from various aquatic environments. Most of these isolates belonged to two genera, namely *Candida* and *Debaryomyces*. These isolates were preliminary tested for their fermentation capabilities by observing gas production in media containing sodium chloride. But the production of ethanol was not reported. Limtong *et al*. (1998) hybridized *Saccharomyces cerevisiae* M30, a high ethanol producing strain, with *Zygosaccharomyces rouxii* TISTR1750, a halo-tolerant strain, using polyethylene glycol–induced protoplast fusion. Compared with the parental strains, one of the derived strains (Fusant RM11) exhibited higher ethanol producing capacity in terms of both ethanol concentration and ethanol yield, in salted glucose broth media containing 1.5%, 3%, 5% or 7% sodium chloride. Using the medium containing 18% glucose and 3% sodium chloride, the Fusant RM11 showed maximal ethanol production of 68.5 g L^−1^, while the parental strains, *S. cerevisiae* M30 and *Z. rouxii* TISTR1750, produced 65.0 and 63.6 g L^−1^ bioethanol, respectively. The fermentations were carried out at 30 °C for 60 h.

Kathiresan *et al*. (2011) isolated 10 marine yeast strains from mangrove sediments on the south-east coast of India. These isolated strains were *Candida albicans*, *Candida tropicals*, *Debaryomyces hansenii*, *Geotrichum* sp., *Pichia capsulata*, *Pichia fermentans*, *Pichia salicaria*, *Rhodotorula minuta*, *Cryptococcus dimennae* and *Yarrowia lipolylica*. They reported that *Pichia salicaria* was the best strain for ethanol production with 12.3 ± 0.8 g L^−1^ bioethanol from sawdust filtrates at 2% concentration after 120 h of incubation. When 2% sawdust hydrolysis (hydrolysed by dilute phosphoric acid) as the carbohydrate source, 26.2 ± 8.9 g L^−1^ bioethanol was produced by *P. salicaria*. Later, Senthilraja *et al*. (2011) reported that in fermentations using free cells, *P. salicaria* produced the highest ethanol concentration of 28.5 ± 4.32 g L^−1^ among these 10 isolates. When these yeast cells were immobilized in sodium alginate, improved ethanol production was observed in fermentations using all strains. *Candida albicans* exhibited the highest ethanol production of 47.3 ± 3.1 g L^−1^.

Obara *et al*. (2012) studied the bioethanol production from the hydrolysate of paper shredder scrap using a marine yeast isolated from Tokyo Bay. It was found that the marine yeast – *S. cerevisiae* – (strain C-19) showed high osmotic tolerance and high ethanol production. It produced 122.5 g L^−1^ of ethanol from a medium containing 297 g L^−1^ of glucose. The maximum bioethanol concentrations for the control strains, *S. cerevisiae* NBRC 10217 and *S. cerevisiae* K-7, were 37.5 and 98.5 g L^−1^, respectively. Moreover, the fermentation using the marine yeast C-19 reached peak ethanol production at day 3, while both control strains required 7 days to achieve their maximum bioethanol production. The high osmotic tolerance of the marine yeast strain was considered to contribute to its promising performance. As this strain belongs to *S. cerevisiae* species, it could be amenable to the existing genetic modification tools that developed on the basis of *Saccharomyces* sp. for further improvement.

Saravanakumar *et al*. (2013) compared bioethanol production using a marine *S. cerevisiae* strain and a terrestrial *S. cerevisiae* strain. In fermentations using the hydrolysate of sawdust as the substrate, the marine strains showed maximum ethanol production of 25.1 g L^−1^, while the terrestrial strain produced only 13.8 g L^−1^ ethanol.

Khambhaty *et al*. (2013) isolated a marine yeast strain (*Candida* sp.) from Veraval, on the west coast of India. This strain was able to convert galactose, sugar cane bagasse hydrolysate as well as the hydrolysate of a red seaweed *Kappaphycus alvarezii* into bioethanol under a wide range of pH (2.0–11.0) conditions and in the presence of high concentration of salts (2.5–15% w/v). Sugar cane bagasse hydrolysates were prepared using H_2_SO_4_ and HCl, resulting in 7.17% and 7.57% reducing sugar, respectively. Around 22.8 and 18.9 g L^−1^ ethanol were obtained, equating to conversion efficiencies of 66% and 55%, respectively. In a seaweed hydrolysate containing 5.5% reducing sugar with 11.25% salt concentration, around 12.3 g L^−1^ ethanol was produced after 72 h of incubation, representing 50% conversion efficiency. When the seaweed hydrolysate was diluted by freshwater with a ratio of 3 : 1 or 1 : 1, 100% carbohydrate conversions were observed within 48 h. Moreover, *c. *21–24 g L^−1^ bioethanol was produced in fermentation using a GYE broth media containing 5% galactose in the presence of 0–10% of KCl, CaCl_2_ and NaCl.

Khambhaty *et al*. (2013) concluded that the presence of 2–13% salt benefited the growth of their isolate. Although fermentation efficiency was relatively low in a medium containing 11.25% salt, 100% fermentation efficiency could be achieved in fermentations using media containing 6.25–9% salt. Their isolate could also tolerate a wide range of pH from 4 up to 10 with very little growth difference. They claim that the pH and salt tolerance of the marine yeast made it a promising candidate for fermentations under different environmental conditions. Khambhaty's findings were in line with a study conducted by Gupta (1996) who reported that various species of yeasts such as *Debaryomyces*, *Rhodotorula*, *Candida* and *Saccharomyces* could tolerate high concentration of NaCl up to 16%. In addition, yeasts that could tolerate NaCl up to 3.5 M (20.5%) have also been reported (Kutty & Philip, 2008).

Various biological materials have been investigated for the generation of bioethanol, such as wheat straw (Pensupa *et al*., 2013), sugarcane bagasse (Chandel *et al*., 2013) and corn stover (Bondesson *et al*., 2013). Recently, various marine biomass sources, for example seaweed (Khambhaty *et al*., 2013) and sea lettuce (Yanagisawa *et al*., 2011), have attracted increasing attention as a promising nonfood material for bioethanol production, as they do not compete with edible crops in terms of land and freshwater resources. The hydrolysis of marine biomass could result in a salty hydrolysate, which would require desalting (e.g. electrodialysis) before fermentation when terrestrial yeasts are used (Mody *et al*., 2013). However, halophilic yeasts, especially yeasts isolated from a marine environment, would be able to directly ferment the salty hydrolysate to bioethanol (Khambhaty *et al*., 2013). Therefore, the energy intensive step, desalting, could be avoided, making the whole fermentation process more economically competitive (Khambhaty *et al*., 2013). Table1 compares the bioethanol produced using various yeast strains isolated from the marine environment and their respective fermentation conditions.

**Table 1:** Ethanol production by marine yeasts

References	Yeast name	Isolation source	Substrate	Hydrolysis method	Fermentation condition (sugar conc., temp., incubation time)	Ethanol conc. g L^−1^
Khambhaty *et al*. (2013)	*Candida* sp.	Veraval, the West coast of India	Seaweed	2.5% H_2_SO_4,_ cooked at 100 °C for 1 h	3.77% sugar, 30 °C, 48 h	17.6
							Sugarcane Bagasse	2.28% sugar, 30 °C, 48 h		7.7*
						Galactose	N/A	5% galactose, 30 °C, 0–10% of KCl, 24 h	21–24
Saravanakumar *et al*. (2013)	*S. cerevisiae*	Mangrove soil, southeast coast of India	Sawdust	0.8% H_3_PO_4_	6.84 mg L^−1^ sawdust, 30 °C, 89 h	0.0024*
Obara *et al*. (2012)	*S. cerevisiae*	Tokyo Bay, Japan	Paper shredder scrap	3% H_2_SO_4_ at 121 °C for 1 h then enzymatic saccharification	29.7% of glucose from paper shredder scrap, 30 °C, 72 h	122.5
										Enzymatic saccharification only (cellulase for 2 days at 50 °C and 150 r.p.m.)		
									Immobilized	13
						Sawdust	NaOH 4% at 121 °C for 30 min	2% of sawdust, 28 °C, 120 r.p.m. for 72 h	7.6
Kathiresan *et al*. (2011) Senthilraja *et al*. (2011)	*Candida albicans*, *C. tropicals*, *D. hansenii*, *Geotrichum* sp., *Pichia capsulata*, *Pichia fermentans*, *Pichia salicaria*, *R. minuta*, *C. dimennae* and *Y. lipolytica*	Sediments, southeast coast of India	Glucose	N/A	28 °C, 120 r.p.m. for 96 h. Nonimmobilized 28 °C, 120 r.p.m. for 96 h. Immobilized	9.8–28.5 13–47.3
								Sawdust	NaOH 4% at 121 °C for 30 min	2% of sawdust, 28 °C, 120 r.p.m. for 72 h	1.7–12.3

* No ethanol concentration was reported in the original papers. This value was estimated based on the conversation efficiencies reported by the references.

The recent research has shown great potential of marine yeasts in bioethanol production. But more investigation should be conducted to further demonstrate the benefits of using marine yeasts in bioethanol industry, especially in bioethanol fermentations using marine biomass based substrate. Subsequently, more marine yeasts should be isolated to explore their potential. The isolates should be selected based on their capability of utilizing and fermenting a wide range of sugars that presented in marine biomass hydrolysate, including galactose, xylose, mannitol and fucose. Also, the isolates should have high tolerance capacity to salts and inhibitors that may be generated during the hydrolysis of marine biomass.

## Use of marine yeast for the production of pharmaceutical products

Several terrestrial yeasts, such as *S. cerevisiae* and *Pichia pastoris*, have been developed as hosts for the commercial production of pharmaceutical proteins, such as insulin, hepatitis B vaccines and caseinomacropeptide (Du & Webb, 2011). Similarly, several marine yeast species, such as *Yarrowia* sp. and *Candida* sp. have been reported to be able to synthesize different pharmaceutical products, as shown in Table2. In comparison with terrestrial yeasts, the investigation on marine yeasts for the pharmaceutical production is still at its early stage. This review will discuss three examples; astaxanthin, siderophore and riboflavin, as they are the most widely explored pharmaceutical products that could be produced using marine yeasts.

**Table 2:** Products of industrial marine yeast and their applications

Products	Marine yeasts species	Applications	Source
Fuels			
Bio-ethanol	*Candida albicans*, *Candida tropicals*, *Debaryomyces hansenii*, *Geotrichum* sp., *Pichia capsulata*, *Pichia fermentans*, *Pichia salicaria*, *Rhodotorula minuta*, *Cryptococcus dimennae* and *Yarrowia lipolytica*	Biofuel industries	Kathiresan *et al*. (2011)
			*Saccharomyces cerevisiae* C-19		Obara *et al*. (2012)
			*Candida* sp.		Khambhaty *et al*. (2013)
Microbial oil	*Rhodotorula mucilaginosa*	Biodiesel industries	Li *et al*. (2010a, b)
			*Yarrowia lipolytica*		Katre *et al*. (2012)
Pharmaceuticals			
Alginate lyase	*Yarrowia lipolytica*	Pharmaceutical industries	Liu *et al*. (2009)
Exo-β-1,3 glucanase	*Williopsis saturnus* WC91-2	Pharmaceutical industries	Peng *et al*. (2009, 2011)
Riboflavin	*C. membranifaciens* subsp. *flavinogenie*	Food and pharmaceutical industries	Wang *et al*. (2008)
	*Candida tropicalis*		Amornrattanapan (2013)
Silver nanoparticles	*Yarrowia lipolytica* NCYC 789	Antimicrobial	Apte *et al*. (2013)
Copper-zinc superoxide dismutase	*Debaryomyces hansenii*	Anticancer	Hernandez-Saavedra & Ochoa (1999)
Industrial enzymes			
Lipase	*Aureobasidium pullulans*	Chemical industries	Zhang & Chi (2007)
	*Leucosporidium scottii* *Cryptococcus adeliensis*		Duarte *et al*. (2013)
Cellulase	*Aureobasidium pullulans*		Chi *et al*. (2009)
	*Pichia salicaria*		Kathiresan *et al*. (2011)
Inulinase	*Pichia guilliermondii*	Food and fuel industries	Chi *et al*. (2009)
Acid protease	*Metschnikowia reukaufii* W6b	Food and pharmaceutical industries	Li *et al*. (2010a, b)
Protease	*Rhodotorula mucilaginosa*	Feed industries	Duarte *et al*. (2013)
α Glucosidases	*Leucosporidium antaracticum*	Pharmaceutical industries	Turkiewicz *et al*. (2005)
Endoxylanase	*Candida davisiana*, *Cryptococcus adeliensis*, *Guehomyces pullulans*	Chemical industries	Duarte *et al*. (2013)
Phytase	*Kodamea ohmeri*	Feed industries	Li *et al*. (2008)
Other products			
Silver nanoparticles	*Candida albicans*, *C. tropicals*, *Debaryomyces hansenii*, *Geotrichum* sp., *Pichia capsulata*, *Pichia fermentans*, *Pichia salicaria*, *Rhodotorula minuta*, *Cryptococcus dimennae* and *Yarrowia lipolylica*	Biomaterial industry	Subramanian *et al*. (2010)
Nanoparticles	*Yarrowia lipolytica*		Agnihotri *et al*. (2009)
Degrader of pollutants	*Yarrowia lipolytica*	Bioremediation	Bankar *et al*. (2009)
Degrader of pollutants	*Yarrowia lipolytica*	Hydrocarbon degradation	Oswal *et al*. (2002)
Viable cells	*Yarrowia lipolytica*	Bioremediation of TNT-polluted marine environments	Jain *et al*. (2004)
Single-cell protein	*Cryptococcus aureus*, *Yarrowia lipolytica*	Food and feed industries	Zhang *et al*. (2009a, b)
	*Cryptococcus aureus* G7a		Gao *et al*. (2007a, b)
Carotene	*Rhodotorula* sp.	Food colouring	Cong *et al*. (2007)
	*Rhodotorula mucilaginosa*, *Arxula adeninivorans*		Libkind *et al*. (2004)

Astaxanthin is a carotenoid compound, responsible for the orange-red colour of some living organisms. It is the main carotenoid used in the aquaculture industry worldwide (Higuera-Ciapara *et al*., 2006). It has been reported that astaxanthin has a wide range of pharmacological properties including antioxidant and antimicrobial activity and reducing risk of certain cancers and cardiovascular diseases (Neuman *et al*., 1999; Ushakumari & Ramanujan, 2013). In addition, astaxanthin can enhance the immune response to viral, bacterial, fungal and parasitic infections as well as reducing the risk of cataracts, atherosclerosis and macular degeneration (Cooper *et al*., 1999).

Roche began the large-scale production of synthetic astaxanthin in 1990. However, an ever-growing demand for natural foods and the high cost of synthesizing the pigment have stimulated research into alternative natural sources of astaxanthin (Higuera-Ciapara *et al*., 2006). A marine yeast strain identified as *Rhodotorula glutinis* YS-185, isolated from the Pacific Ocean by He and his team, was found to be capable of producing astaxanthin (He *et al*., 2011). The optimum fermentation conditions for astaxanthin production were 25 °C, using a medium consisting of only glucose (8 g L^−1^) and peptone (8 g L^−1^) with an initial pH of 5.5. Temperature was reported to be a key factor for both astaxanthin production and yeast growth. An astaxanthin concentration of 2.67 μg mL^−1^ was achieved, which was 69.7% higher than that before the fermentation optimization. Ushakumari and Ramanujan (2013) isolated a marine yeast strain from the marine sediments collected from Kerala, India. The astaxanthin was extracted and tested for its activity against *Bacillus subtilis*, *Salmonella typhi*, *Staphylococcus aureus* and *Pseudomonas aeruginosa*. The astaxanthin solution extracted from fermentations using their marine yeast isolate exhibited better antibacterial activity than the standard chloramphenicol.

Siderophores are low-molecular-weight ligands (500–1000 Da), which have extremely high affinity as iron-chelating agents. They are synthesized by many microorganisms during extreme iron deficiency conditions to facilitate the solubilization of extracellular ferric iron (Winkelmann, 2002; Wang *et al*., 2009). Siderophores have wide medical, agricultural and environmental applications (Renshaw *et al*., 2002). For example, the ability of using siderophores could give the microorganism a competitive advantage over other microorganisms as the siderophore transport system will enable the microorganism to compete effectively for the available irons (Renshaw *et al*., 2002; Murugappan *et al*., 2012). Siderophores was also used to control the growth of some pathogenic bacteria isolated from infected marine fish (Renshaw *et al*., 2002).

Wang *et al*. (2009) isolated more than 300 yeast strains from different marine environments and screened them for their abilities to produce siderophore. Among these isolates, only one strain was found to produce high level of the siderophore. This strain was identified as *Aureobasidium pullulans* (black yeast). Under optimal conditions, the strain could produce 1.1 mg mL^−1^ of crude siderophore. The crude siderophore extract was able to inhibit the growth of *Vibrio anguillarum* and *Vibrio parahaemolyticus* that were isolated from sick marine animals.

In similar research, Murugappan *et al*. (2012) obtained 0.7 mg mL^−1^ crude siderophore after 134 h of fermentation using a marine yeast strain (*A. pullulans*). This strain was isolated from seaweed (*Ulva fasciata*) samples that were collected from the coastal region of the Gulf of Mannar, India.

Riboflavin (vitamin B2) is required by all bacteria, plants, animals and human beings. It serves as a precursor for two coenzymes: flavin mononucleotide and flavin adenine dinucleotide. Riboflavin can be synthesized only by plants and microorganisms, while human and other animals must acquire it from their diets (Chi *et al*., 2008). Although chemical synthesis processes still dominate the market, the biosynthesis of riboflavin offers several distinctive advantages, such as lower energy needs, less chemical waste generated and easier recovery (Stahmann *et al*., 2000).

So far several terrestrial yeast species have been investigated for the riboflavin biosynthesis, such as *Pichia guilliermondii* (Leathers & Gupta, 1997; Faiura *et al*., 2007) and *Candida famata* (Stahmann *et al*., 2000; Boretsky *et al*., 2005). Leathers and Gupta (1997) found that *P. guilliermondii* could produce up to 14.4 μg mL^−1^ of riboflavin when xylose was used as the carbon source, while no more than 5.1 μg mL^−1^ of riboflavin could be produced when glucose replaced the xylose. Furthermore, Boretsky *et al*. (2005) showed that some types of yeast (such as *C. famata* and *P. guilliermondii*) can only overproduce riboflavin in media deficient in iron because iron represses riboflavin synthesis.

Wang *et al*. (2008) isolated a marine yeast strain from seawater from the China Eastern Sea, which was identified as *Candida membranifaciens* subsp. *flavinogenie*. This strain produced 16.3 μg mL^−1^ of riboflavin using an iron-deficient medium, while it produced only 0.1 μg mL^−1^ of riboflavin when 0.005% FeCl_3_ was added. Further investigation showed that the riboflavin synthesis was enhanced by vigorous shaking during cultivation in medium containing galactose, maltose, sucrose or xylose. Around 22 μg mL^−1^ of riboflavin was achieved within 54 h of fermentation under these optimal conditions. A recent study carried out by Amornrattanapan (2013) isolated 47 marine yeast strains for the screening of their capacity of riboflavin production. Among those isolates, the best strain *Candida tropicalis* MICBUU002 produced 254.22 μg mL^−1^ riboflavin, which was more than 10-fold higher than that reported by Wang *et al*. (2008). The strain was cultured at 30 °C for 5 days using 2% glucose as the only carbon source.

## Use of marine yeast for industrial enzymes production

As for terrestrial yeasts, marine yeasts (e.g. *Aureobasidium* sp. and *Pichia* sp.) have also been investigated for the production of enzymes, such as inulinase (Chi *et al*., 2009), amylase (Li *et al*., 2007a,  b), superoxide dismutase (Ramirez-Orozco *et al*., 1998), and lipase (Chi *et al*., 2009). As marine yeasts live in high salinity environments, these enzymes are expected to have distinct properties, for example high salt tolerance, thermostability, barophilicity and cold adaptivity (Sarkar *et al*., 2010). In this mini review, inulinase and amylase were used as examples for the demonstration of marine yeast–based enzyme production. Other case studies were listed in Table2 and parts of them were also described by Chi *et al*. (2009) and Sarkar *et al*. (2010).

Inulinase catalyses the hydrolysis of inulin to fructose. Inulin is found in many types of plants, such as Jerusalem artichoke, dahlia tubers or chicory root (Baheri *et al*., 2001; Liu *et al*., 2003; Sarkar *et al*., 2010), it is a polymer formed of linear (β-1,2)-linked fructose. It could be used as food additive in food applications, or as feedstock in the biofuel and pharmaceutical industries (Chi *et al*., 2011). Inulin can be hydrolysed chemically; however, the chemical process is associated with the formation of undesired by-products, such as di-fructose anhydrides (Gill *et al*., 2006), while enzymatic hydrolysis of inulin yields 95% pure fructose (Gao *et al*., 2007a,  b; Bharathi *et al*., 2011).

Many yeast species can produce inulinases, including *Candida* sp., *Kluyveromyces* sp., *Pichia* sp. and *Sporotrichum* sp. (Pandey *et al*., 1999). Gao *et al*. (2007a,  b) screened out four marine yeast strains from 427 yeast isolates obtained from different marine habitats, including guts of marine fish, marine algae, seawater, sea sediments and salterns, for the production of inulinase. These strains were identified as *P. guilliermondii*, *Cryptococcus aureus*, *Yarrowia lipolytica* and *D. hansenii*. A maximum inulinase activity of 62.85 U mL^−1^ was recorded in the fermentation using the yeast strain *Y. lipolytica*. However, no mono- or disaccharides were detected after inulin hydrolysis using crude inulinase produced by *Y. lipolytica*, indicating that this crude inulinase had no exo-inulinase activity. High exo-inulinase activity was, however, detected in the crude enzyme extract obtained from the fermentation of *P. guilliermondii*, *C. aureus* and *D. hansenii* strains (Gao *et al*. 2007a,  b). Later, the inulinase gene of the marine yeast *P*. *guilliermondii* was successfully cloned and expressed in *P. pastoris* X-33 (Zhang *et al*., 2009a,  b). The inulinase activity achieved in fermentations using the recombinant strain reached 286.8 U mL^−1^. Also, high exo-inulinase activity was detected in the purified recombinant enzyme (Zhang *et al*., 2009a,  b). The inulin hydrolysate, catalysed by the recombinant inulinase, was converted into bioethanol using *Saccharomyces* sp., and the ethanol concentration achieved was 140 g L^−1^ (Wang *et al*., 2013). Although inulinase production has not been commercialized, these findings indicated that the recombinant inulinase might have potential to be used in the biofuel industry in the future.

Bharathi *et al*. (2011) isolated a marine yeast strain (SY3) from the gut of the fish *Lutjanus campechanus* and identified it as *Cryptococcus* sp. Fermentations with the SY3 strain were carried out in shake flasks using YPD media. After partial purification of the enzyme solution by dialysis, the inulinase activity reached 62.7 U mL^−1^. The optimal condition for inulinase production by this strain was pH 4.0 and 37 °C using a medium consisting of 4.0% (w/v) inulin and 0.5% (w/v) yeast extract.

Amylases have been widely used in baking, food, pharmaceutical, detergent, textile and biofuel industries for the hydrolysis of starch. It has been reported that several terrestrial yeasts are able to produce extracellular amylolytic enzymes, for example *Arxula adeninivorans*, *Candida japonica* and *Saccharomycopsis fibuligera* (Chi *et al*., 2003). Similarly, Li *et al*. (2007a,  b) have investigated crude glucoamylase production using a marine yeast strain *A. pullulans* N13d, which was isolated from the deep sea of the Pacific Ocean. It was found that the highest amylase yield was achieved in the late stationary phase of cell growth. Within 56 h of fermentation, 58.5 units mg protein^−1^ of amylase were produced under optimal condition (Li *et al*., 2007a,  b). This enzyme was tested for its ability to hydrolyse potato starch, raw corn starch and sweet potato starch, respectively. The amylase demonstrated good hydrolytic ability on potato starch (with a yield of 68.5% within 6 h) but not on corn or sweet potato starch (Li *et al*., 2007a,  b). In a solid-state fermentation using a marine yeast *S. fibuligera* A11, an amylase activity of 4296 U g^−1^ of dry substrate was obtained (Chen *et al*., 2010). The substrate contained 610.0 mL kg^−1^ moisture, 30.0 mL kg^−1^ inoculums (OD_600_ = 20.0), wheat bran to rice husk ratio 0.42, cassava starch 20.0 g kg^−1^. Then, *S. fibuligera* was immobilized and co-cultured with oleaginous yeast *Rhodosporidium toruloides* (Gen *et al*., 2014). The yeast strain produced amylase, which converted cassava starch to glucose, while the oleaginous yeast consumed the resulting glucose to accumulate single-cell oil. In 2-L scale fermentation, a single-cell oil yield of 64.9% (w/w) was obtained from a medium containing 60 g L^−1^ cassava starch.

Besides bioenergy, pharmaceutical and enzyme production, marine yeasts have also showed potential to be utilized in various other fields, such as synthesis of metal nanoparticles (Turkiewicz *et al*., 2005; Li *et al*., 2010a,  b), degradation of pollutants (Agnihotri *et al*., 2009; Bankar *et al*., 2009; Subramanian *et al*., 2010) and use as a probiotics in marine animal culture. Several case studies are summarized in Table2.

## Conclusions

Marine yeasts live in harsh environments, which provide the potential for several unique desirable properties to be used in various industries. The latest development in the methodology of marine yeast isolation and cultivation offers the opportunity of discovering novel marine yeasts. Various media have been proposed by different research groups to suit for the different requirement of marine yeasts. These media are rich in nutrients, and they are common to contain antibiotics to reduce the bacterial and mould contamination. Using marine yeasts in bioethanol production shows distinctive advantage on the osmosis tolerance, the possibility of utilization of seawater instead of fresh water and the potential of advantage in using marine biomass as a substrate. Marine yeasts have already been investigated for the production of pharmaceutical and enzymatic products, such as astaxanthin, siderophore, riboflavin, inulinase and amylases. Yet, the commercial application of marine yeasts is still limited. The current research, however, indicates the promising features of the marine yeasts for the potential industrial application and their superiority over the terrestrial ones in certain field. More direct comparison studies should be carried out to give further evidence on the advantages of marine yeasts over terrestrial yeasts.
